# Complex Regional Pain Syndrome in a Non-traumatic Case: A Case Report

**DOI:** 10.7759/cureus.62812

**Published:** 2024-06-21

**Authors:** Yacoub Abuzied, Mohammad Jaber, Mona Hafiz, Rana Al-Hamwy

**Affiliations:** 1 Department of Nursing, Rehabilitation Hospital, King Fahad Medical City, Riyadh, SAU; 2 Department of Nursing, Emergency and Trauma Centre, King Fahad Medical City, Riyadh, SAU; 3 Family Medicine and Employee Health Department, King Fahad Medical City, Riyadh, SAU

**Keywords:** neuropathic pain, shoulder-hand, pain syndrome, non-traumatic crps, complex regional pain syndrome

## Abstract

Complex regional pain syndrome (CRPS) is an uncommon neuropathic pain illness characterized by extreme discomfort, muscular weakness, limb edema, and hyperhidrosis. Fracture, surgery, stroke, and spinal cord damage are all potential risk factors. This case report study provides a detailed description of no-traumatic CRPS, a complex pain illness characterized by sensory, vasomotor, sudomotor, motor, trophic, and edematous changes and persistent discomfort. We reported a case of a 39-year-old male with a seven-year history of severe right shoulder-hand pain. He presented with recurrent hand and shoulder pain characterized by burning sensations and weakness, despite not having experienced this before, and he denied any history of trauma or fracture. Surgical procedure and pharmaceutical therapy were provided, but there was no outcome, as evidenced by the patient's clinical condition and the medical records. Despite extensive investigation, no imaging or laboratory tests have been developed for diagnosis, necessitating further research for a comprehensive understanding and diagnosis.

## Introduction

Complex regional pain syndrome (CRPS) is a rare neuropathic pain disorder causing extreme discomfort, muscle weakness, limb edema, and hyperhidrosis, with potential risk factors including fracture, surgery, stroke, and spinal cord damage [1]. Underlying causes include a variety of processes, including sensory, motor, inflammatory, immune/auto-immune, autonomic, and genetic impacts [2]. The main characteristics of CRPS include spontaneous pain, hyperalgesia, allodynia, and abnormal vasomotor and sudomotor activity [3].

The syndrome was characterized by two types: CRPS type 1, formerly known as reflex sympathetic dystrophy (RSD), and CRPS type 2, formerly known as causalgia. While both types are often caused by trauma, the primary differentiating feature is the existence of distinct nerve damage, which is missing in type 1 but present in type 2 CRPS [4].

CRPS can develop without an evident injury or due to prolonged immobility [5]. Things that could increase the risk of having CRPS are poor nerve health, such as diabetes, which may cause weakness and impair the nerves' ability to repair themselves. In this case, the patient had a free past medical history and was not diabetic but used the computer for extended periods [5].

Hyperalgesia and allodynia are the hallmarks of CRPS, a chronic pain disorder that typically affects the limbs. It usually appears following surgery or trauma to the extremities [1].

In this case, the patient had shoulder-hand pain for almost seven years associated with severe pain, burning sensations, and tension, which increased mainly with prolonged computer use and/or heavy exercise and tasks. Upon investigation, the patient was free from arthritis, broken bones, nerve entrapment, dislocation, and tendinitis. However, because no particular diagnostic tests are available for this illness, healthcare professionals frequently rely on clinical data to diagnose it [6].

This case report aims to highlight an important and complicated pain issue that persisted, causing severe discomfort, with no clear diagnostic tests or procedures for such cases.

## Case presentation

A 39-year-old male with a free past medical history presented to the Employee Health and Staff clinic complaining of recurrent hand and shoulder pain, which was an old issue for him as he denied any history of trauma or fracture. With a seven-year history of severe right shoulder-hand pain characterized by severe burning sensations and tension, this condition was new to him. It had not occurred before, according to the patient, as he is a senior nurse who was able to characterize and describe the symptoms and nature of pain as symptoms persisted. Seven years ago, following an in-depth medical examination, there was sudden hand swelling characterized by a severe burning sensation and tension at the right elbow and biceps muscle, with a severe intermittent burning sensation over the wrist area during computer use or prolonged hand use activities. The symptoms were treated with a dexamethasone course with dosage tapering for 14 days in addition to specific pain medications and muscle relaxants, where the hand swelling improved slightly after the therapeutic course while the pain remained the same with the same previous symptoms. Later on, after several diagnostic tests and procedures such as magnetic resonance imaging (MRI), computerized tomography (CT), and nerve conduction studies (NCS), the status ended with an ulnar nerve exploration to explore the ulnar nerve for further interventional assessment and evaluation, which ended with no surgical intervention as there was no clear diagnosis till date. Later on, after the surgical procedure, pharmaceutical therapy, and recovery period, the patient effectively dealt with his illness by adopting effective coping mechanisms along with pain medications and muscle relaxants as needed. In addition, adopt a healthier lifestyle and exercise as recommended by physiotherapists. Specific diagnostic tests were repeated recently to re-diagnose the case as per the patient's request, as he had again severe pain that affected his lifestyle, which revealed no changes to his condition.

Diagnostic assessment

Several diagnostic tests and procedures were done to diagnose the case: a routine chest X-ray, a radiologic examination of a bilateral upper limb X-ray, a bilateral wrist joint X-ray, and a right shoulder X-ray, all of which showed normal findings in 2017. Further, the neck's MRI showed a degenerative disc disease at C3-C4, C4-C5, C5-C6, and C6-C7 levels. A shoulder MRI showed no rotator cuff tendinopathy and minimal subacromial subdeltoid bursitis. Additionally, an ultrasound examination of the right arm revealed a performance similar to the normal contralateral side. It demonstrates the minimal difference in the biceps muscle in the form of a contracted, bulky right muscle compared to the left one. However, there was no mass syndrome, muscle injury, or echo structure abnormality; the biceps tendon and unremarkable subcutaneous tissue appeared normal; the right upper limb veins showed no sonographic evidence of acute venous thrombosis in the right upper limb veins; and there was no evidence of flow-limiting stenosis in the right upper limb arteries. The left arm was examined, and it was found to have normal findings.

The patient was referred to a pain management clinic for further follow-up and opinion; they decided to go for a dexamethasone injection at the right shoulder due to minimal subacromial subdeltoid bursitis and for the degenerative disc, specifically for C5-C6. The patient was injected with dexamethasone in the operating room and rested for three days to allow for recovery. The patient claimed that there was severe pain at the site of the injection, and there were no changes to his condition even after the recovery period. As the issue persisted, a NCS was done to explore the pain. The NCS showed normal findings, as there was no electrodiagnostic evidence of large-fiber neuropathy.

A referral by the neurological and spine surgeon to the neurosurgery specialty for further assessment and follow-up as the issue persisted which led the neurosurgery team to go for another NCS to explore the chronic right forearm/hand discomfort. Referring to the patient history, there was decreased sensation at the right median nerve distribution, abductor pollicis brevis (APB) power 4/5. Another NCS is needed to confirm the diagnosis of right median neuropathy. Later on, nerve conduction velocity (NCV) and electromyography (EMG) findings showed that all nerve conduction studies were within normal limits. All examined muscles showed no evidence of electrical instability. This electrodiagnostic study failed to show electrophysiological evidence of distal or proximal median nerve lesions. There was also no evidence of brachial plexopathy or cervical radiculopathy. In early 2018, referring the results to the neurosurgery team, they decided to go for an ulnar nerve exploration and decompression procedure, as advised by the neurosurgeon, for further assessment. One week later, the patient was admitted for an ulnar nerve exploration procedure under general anesthesia (GA), which showed normal findings.

In contrast, direct NCS to the ulnar nerve was done in the theater and showed no difference in the readings of the ulnar nerve activity before the procedure and no changes following the surgical procedure. Rest was given for six weeks to allow the wound to heal and for recovery, whereas an arm sling was applied with a monthly follow-up to the clinic. After another follow-up to the clinic three months later, upper limb computed tomography angiography (CTA) was done for further assessment, which showed an unremarkable right upper limb angiogram and no abnormal aneurismal dilatation of the right upper limb arteries.

After completing all imaging procedures, another NCS and a visit to the neurosurgery clinic were scheduled for further assessment and evaluation. Later on, the patient was referred to physiotherapy (PT) to allow physical rehabilitation of the nerves and muscles for further assessment and evaluation. Extensive rehabilitation sessions and follow-ups were done, but the patient and the PT observed no progress on the status. Consequently, a referral for a course of dry needles was made to the shoulder and the back through the pain management clinic, but nothing was changed to the patient's condition as the symptoms remained the same.

Three months later, the patient stated that he started to adopt a healthier lifestyle and engaged in regular exercise as he began to adjust his life by avoiding strenuous exercise and heavy computer use. There was a slight improvement, according to the patient, but with special precautions and safety measures.

**Figure 1:**
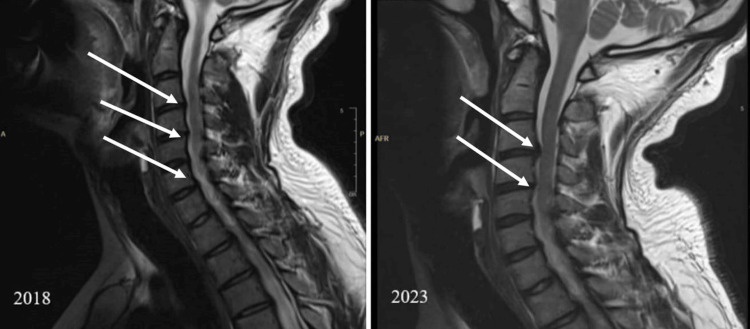
Cervical spine MRI comparison 2018 and 2023 showed a degenerative disc disease at C3-C4, C4-C5, C5-C6, and C6-C7 levels and cervical spondylosis.

**Figure 2:**
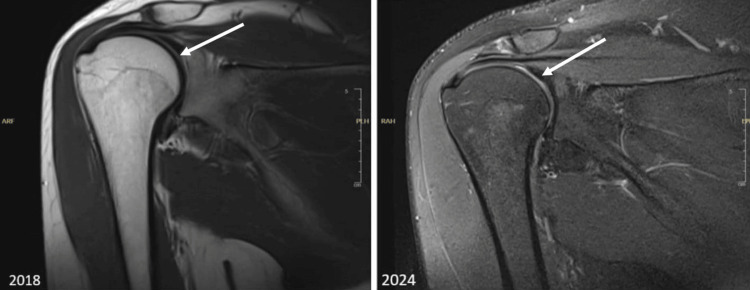
Right shoulder MRI comparison 2018 and 2024 showed no rotator cuff tendinopathy and minimal subacromial subdeltoid bursitis and minimal supraspinatus tendinosis, and no rotator cuff tear.

The patient reported back to the staff clinic in 2023 with the same previous symptoms described, as he wanted to fix the issue and follow up on it. A cervical spine MRI was done and showed cervical spondylosis, and a right shoulder MRI showed minimal sub-acromial sub-deltoid bursitis, minimal supraspinatus tendinosis, and no rotator cuff tear. There was no difference between the initial MRI, which was in 2018, and the one done earlier in 2023 (Figures 1-2).

**Figure 3:**
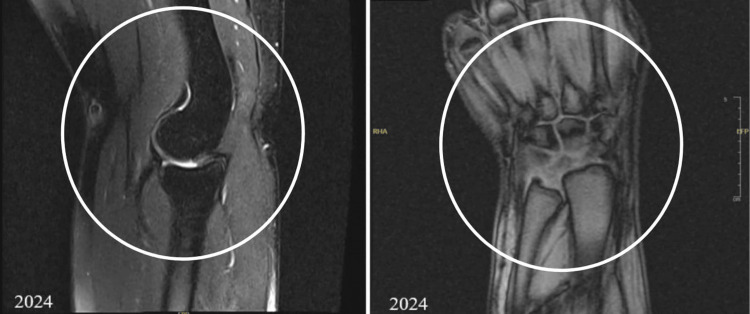
Right elbow and wrist MRI, elbow MRI showed no joint effusion or erosions. Mild sprain at the ulnar collateral ligament and the fibers are intact. The wrist MRI showed a trace amount of fluid in the distal radial ulnar joint with abnormal signal intensity at the radial side of the triangular fibrocartilage disc which may suggest a tear and there was negative ulnar variance.

In early 2024, an MRI of the right wrist and elbow was done to track the pain and showed no joint effusion or erosions, as there was no previous comparison before this image. Mild sprain at the ulnar collateral ligament, and the fibers are intact. While the wrist MRI was severely degraded by motion, which was due to the positioning and prolonged procedure timing, limiting the assessment of ligaments and tendons, there was also a trace amount of fluid in the distal radial ulnar joint (DRUJ) with abnormal signal intensity at the radial side of the triangular fibrocartilage disc, which may suggest tearing. There was a negative ulnar variance (Figure 3). The patient stated that he was having severe pain during the procedure due to the prone position and extended arm, as he was not able to tolerate the pain due to the lengthy procedure and position. The patient was referred to interventional radiology for further assessment and intervention.

## Discussion

The production of pro-inflammatory modulators is assumed to indicate the two phases of the clinical course of CRPS: the acute, or warm, phase and the chronic, or cool, phase, which are indicated by the activation of fibroblasts, osteocytes, and keratinocytes [7]. Impaired neuropeptide signaling and a pro-inflammatory immune response are two features of CRPS [8]. Since CRPS lacks identifiable histopathologic or biochemical indicators, a clear and accurate description is required. CRPS is frequently diagnosed by exclusion, relying mainly on clinical examination and bone scans [9]. When seeking medical attention for shoulder pain, it is important to assess its location, nature, severity, onset, and duration, as well as any potential risk factors. A history of malignancy, a recent infection, or trauma should alert doctors that red flags might be present [10].

Traumatic CRPS usually occurs following an injury, surgery, stroke, or heart attack. On the other hand, non-traumatic CRPS is more likely to occur during times of severe exhaustion and emotional stress [11]. The pain is disproportionate to the extent of the initial damage. CRPS is uncommon, and its cause remains not well-understood. Treatment is most effective when started early. In such cases, there is an opportunity for improvement or maybe remission [1]. Moreover, there was a strong correlation between the image of the afflicted limb and pain associated with movement; hence, the most prevalent long-term characteristics of CRPS continue to be its clinical manifestations of pain and motor dysfunction [12]. However, MRI cannot differentiate between people with CRPS and those without, since its primary goal in treating suspected cases of CRPS is to rule out other conditions that could better explain the patient's symptoms [13].

In this case, finding the causes and the right solution are important. MRI cannot distinguish between CRPS and non-traumatic CRPS cases because the role of MRI in patients with suspected CRPS is to exclude alternative diagnoses that would better explain patients’ symptoms [13]. Because no imaging has been established for the diagnosis of CRPS, imaging studies for arm and upper limb vascular US for arteries and upper limb CTA are secondary diagnostic imaging studies that have not produced concrete evidence of the condition. As a result, recently proposed diagnostic criteria have not yet been validated and are only infrequently used [14].

The patient is still affected by the pain, which is affecting his daily living activities and lifestyle. He has undergone two hospital stays and long sick leaves, as he believes that the procedures were unnecessary and failed to reduce and control the pain, which limited his activities, and impaired his daily life activities. He believes that proper management, guidance, and health education regarding the pain and related symptoms, along with a clear diagnosis, can reduce hospital stays, enhance health services, and improve financial, operational, and clinical outcomes [15]. The patient stated that he was able to adapt to the condition and change his lifestyle. However, this did not completely resolve the issue, as it continues to negatively impact his activities and life, particularly driving, using the computer, and sitting for extended periods. Chronic pain might interfere with regular activities such as working and socializing [16]. It might cause despair, worry, and difficulty sleeping, which can exacerbate discomfort [17].

Adaptive strategies for coping mechanisms are also known as healthy coping strategies and mechanisms. They can improve a person's health and increase emotional resilience [18]. Regular gentle exercises, such as walking or swimming, can help reduce pain over the long term and enhance the nature of the pain [19]. CRPS treatment includes remedial or compensatory approaches or a combination of the two, depending on the particular clinical presentation.

## Conclusions

CRPS exhibits a variety of imaging abnormalities, with sympathetic dysregulation being a significant factor in its development. Imaging modifications, particularly in muscle and bone regions, support this idea. Inflammatory processes are also implicated, with imaging results correlated with acute or chronic inflammatory processes. CRPS can be diagnosed and monitored using imaging, but standardization and consistency in results are needed. Further studies are needed to explore CRPS versus non-traumatic CRPS with regard to shoulder-hand pain syndrome. The most effective therapy choices to address the impairments and functional limits of affected patients with non-traumatic CRPS and shoulder-hand pain are multimodal interventions, which include pharmaceutical treatments, rehabilitation techniques, and lifestyle modifications.
